# Characterization and Phylogenetic Analysis of the Complete Mitochondrial Genome of *Aythya marila*

**DOI:** 10.3390/genes14061205

**Published:** 2023-05-31

**Authors:** Lei Zhang, Tian Xia, Xiaodong Gao, Xiufeng Yang, Guolei Sun, Chao Zhao, Guangshuai Liu, Honghai Zhang

**Affiliations:** College of Life Science, Qufu Normal University, Qufu 273165, China; zhanglei309418@163.com (L.Z.);

**Keywords:** greater scaup, Anatidae, phylogeny, mitochondrial genome

## Abstract

*Aythya marila* is a large diving duck belonging to the family Anatidae. However, the phylogenetic relationship among these *Aythya* species remains unclear due to the presence of extensive interspecific hybridization events within the *Aythya* genus. Here, we sequenced and annotated the complete mitochondrial genome of *A. marila*, which contained 22 tRNAs, 13 protein-coding genes (PCGs), 2 ribosomal RNAs, and 1 D-loop, with a length of 16,617 bp. The sizes of the PCGs ranged from 297 to 1824 bp and were all, except for *ND6*, located on the heavy chain (H). ATG and TAA were the most common start and termination codons of the 13 PCGs, respectively. The fastest- and slowest-evolving genes were *ATP8* and *COI*, respectively. Codon usage analysis indicated that CUA, AUC, GCC, UUC, CUC, and ACC were the six most frequent codons. The nucleotide diversity values indicated a high level of genetic diversity in *A. marila*. *F*_ST_ analysis suggested a widespread gene exchange between *A. baeri* and *A. nyroca*. Moreover, phylogenetic reconstructions using the mitochondrial genomes of all available Anatidae species showed that, in addition to *A. marila*, four major clades among the Anatidae (Dendrocygninae, Oxyurinae, Anserinae, and Anatinae) were closely related to *A. fuligula*. Overall, this study provides valuable information on the evolution of *A. marila* and new insights into the phylogeny of Anatidae.

## 1. Introduction

The greater scaup (*Aythya marila*) is a relatively large diving duck belonging to the order Anseriformes, subfamily Anatinae, and family Anatidae. This species is mainly distributed throughout North Eurasia from Iceland eastward to the Lena River in Siberia, and winters mainly in Northwest Europe and around the Adriatic, Black, and Caspian Seas. In summer, the greater scaup primarily feeds on insects, crustaceans, and mollusks, in particular small bivalves, snails, and amphipods. The greater scaup is the only circumpolar *Aythya* and one of the few circumpolar duck species. It is also the only species in the genus *Aythya* that has subspecies: *A. marila marila* and *A. marila nearctica* [1]. However, to date, little is known about its biology compared to other *Aythya* species, partly due to its relatively isolated breeding grounds and the difficulty in distinguishing it from its close relative, the lesser scaup (*A. affinis*) [2,3].

*Aythya* originated in the Miocene, and there are 12 extant species worldwide [4]. Of these, five species (*A. ferina*, *A. nyroca*, *A. baeri*, *A. fuligula*, and *A. marila*) are widely distributed across China. However, due to the late differentiation between *Aythya* species and serious interspecific hybridization [5,6,7], the phylogenetic relationship among these five *Aythya* species remains unclear. Recent research has shown that there is a substantial spatial overlap in the distribution ranges of *A. nyroca* and *A. baeri*. This overlap may have a detrimental effect on the conservation status of *A. baeri*, which has a relatively small population. As such, it is crucial to assess the potential risk of hybridization between these two species.

Mitochondria are important functional organelles that provide energy for various cellular biological functions [8]. The animal mitochondrial genome is a circular, closed, double-stranded molecule, usually ranging from 15 to 21 kb in size, with 37 genes, including 13 protein-coding genes (PCGs: *ND1*, *ND2*, *ND3*, *ND4*, *ND4L*, *ND5*, *ND6*, *COI*, *COII*, *COIII*, *ATP6*, *ATP8*, and *Cytb*), 2 ribosomal RNAs (12S rRNA and 16S rRNA), 22 transfer RNAs (tRNA), and an A + T-rich noncoding control region [9,10,11]. The mitochondrial genome is a highly conserved molecule characterized by its rapid evolutionary rate, maternal inheritance, haploid nature, and limited recombination in comparison to nuclear genes [12,13,14]. Owing to these attributes, mitochondrial genomes have become increasingly utilized in phylogenetic analyses, population genetic diversity studies, and evolutionary research [15,16,17]. In particular, complete mitochondrial genomes offer substantially more information for phylogenetic analysis than a single mitochondrial gene. However, it is important to note that the mitochondrial genome may not accurately depict the population structure of species due to factors such as gender bias, dispersal behavior, and frequent hybridization events.

In this study, we assembled and annotated the first complete sequence of the mitochondrial genome of the greater scaup and assembled twenty-eight complete mitochondrial genomes of five species: six *A. ferina*, four *A. nyroca*, six *A. baeri*, six *A. fuligula*, and six *A. marila*. We analyzed the gene content, codon usage bias, repeat sequences, and synonymous and nonsynonymous substitution rates of the greater scaup. Additionally, we revealed the phylogenetic relationships among Anatidae species and the population genetic diversity of *A. ferina*, *A. nyroca*, *A. baeri*, *A. fuligula*, and *A. marila* based on a combined set of mitochondrial genes.

## 2. Materials and Methods

### 2.1. Ethics

All experimental protocols were approved by the Qufu Normal University Biomedical Ethics Committee, and all experiments followed the recommendations in the ARRIVE guidelines (Ethical proof 2021097). All animal experiments were performed in accordance with relevant guidelines and regulations.

### 2.2. Specimen Collection, Genome Sequencing, and Assembly

The tissue samples of *A. marila* used in this study originated from the Wildlife Inspection Center of the Northeast Forestry University; no living animal was involved in this study. Samples were stored at −80 °C ultralow-temperature freezer. Total genomic DNA was extracted from the tissue using the DNeasy Blood & Tissue Kit (QIAGEN, Beverly, MA, USA). The whole genome of *A. marila* was sequenced by Sangon Biotech Co., Ltd. (Shanghai, China) using an Illumina NovaSeq 6000 platform (Illumina, San Diego, CA, USA). Approximately 4.7 Gb of raw data from 150 bp paired-end reads were generated. Raw data were processed using Fastp v0.36 [18] and assembled using SPAdes v3.15 [19]. GapFillerr v1.11 [20] was used to fill the gaps, whereas PrInSeS-G was used for sequence correction. In addition, we sequenced 28 samples from 5 species of *Aythya* using an Illumina NovaSeq 6000 platform and obtained high-quality raw genome sequencing data (Table 1). We then used the NOVOPlasty software (V 4.1) to assemble 28 complete mitochondrial genomes of the 5 species: *A. ferina*, *A. nyroca*, *A. baeri*, *A. fuligula*, and *A. marila*.

### 2.3. Annotation and Sequence Analysis

Gene annotation was conducted using the web-based service MITOS (http://mitos.bioinf.uni-leipzig.de/help.py, accessed on 15 August 2022) [21]. The online tRNAscan-SE search server (http://lowelab.ucsc.edu/tRNAscan-SE, accessed on 17 October 2022) [22] was used to annotate the tRNA gene and determine its position and secondary structures were inferred. The mitochondrial genome map of *A. marila* was drawn using the online tool CGView Server (http://cgview.ca/, accessed on 20 October 2022) [23].

The relative synonymous codon usage (RSCU) was calculated using EMBOSS v6.6.0.0 [24], and the base composition was estimated using the BioEdit program (v7.0.9.0) (http://www.mbio.ncsu.edu/BioEdit/bioedit.html, accessed on 21 October 2022) [25]. The Ka/Ks ratios were calculated individually for each protein-coding gene of *A. marila* using DnaSP v65 [26]. GC-skews and AT-skews were used to estimate bias in nucleotide composition across the sequence [27]. The bias of nucleotide composition was calculated using the following formulas [28]:

AT-skew = (A − T)/(A + T) and GC-skew = (G − C)/(G + C)(1)

### 2.4. Phylogenetic Analysis

In this study, we generated two distinct phylogenetic trees to elucidate the evolutionary relationships within the Anatidae family. Initially, to determine the taxonomic position of *A. marila*, we retrieved 51 Anatidae mitochondrial genomes from GenBank and included *Buteo hemilasius* and *Gallus gallus* as outgroup taxa. We constructed Bayesian inference (BI) and maximum likelihood (ML) phylogenetic trees of the 54 mitogenomic sequences based on 13 mtDNA PCGs (*ND1*, *ND2*, *ND3*, *ND4*, *ND4L*, *ND5*, *ND6*, *COI*, *COII*, *COIII*, *ATP6*, *ATP8*, and *Cytb*). MAFFT v. 7.475 [29] was used for the alignment of the nucleotide sequences of the 13 PCGs, and ambiguously aligned regions were identified and removed by Gblocks [30]. The alignments of individual genes were then concatenated using PhyloSuite [31]. According to the Bayesian information criterion (BIC), ModelFinder2.2.1 [32] was used to determine the best partitioning scheme and substitution model, and the GTR + F + I + G4 model was chosen as the best fit for the ML method. ML analysis was performed using IQ-TREE [33] and conducted with 5000 ultrafast bootstrap replicates. The BI method was performed using MrBayes [34] with 4 simultaneous Markov chain Monte Carlo (MCMC) chains running for 2,000,000 generations and sampling every 1000 generations with a burn-in of 25%. Subsequently, to further investigate the population structure among 5 *Aythya* species, we aligned 28 complete mitochondrial genomes using MEGA and constructed a phylogenetic tree, selecting *Anas platyrhynchos* as the outgroup. Lastly, the resulting phylogenetic trees were annotated and visualized using the Interactive Tree of Life (iTOL) online service [35] (https://itol.embl.de/, accessed on 4 October 2022).

## 3. Results and Discussion

### 3.1. Genome Structure and Composition

**Figure 1:**
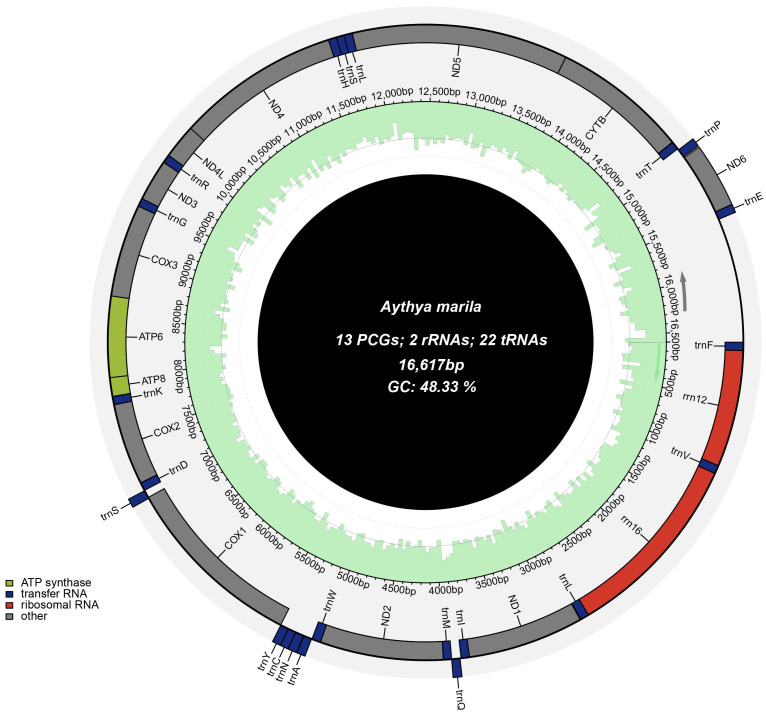
Graphical map of the complete mitochondrial genome of *A. marila*. Genes encoded by the heavy strand are shown inside the circle, whereas those encoded by the light strand are shown outside the circle.

The complete mitochondrial genome sequence of *A. marila* is a typical closed-circular molecule with a size of 16,617 bp and has been deposited in GenBank under the accession number OP326610. The mitochondrial genome contents of *A. marila* are similar to those of most other published *Aythya*, which includes 37 genes [36], 13 PCGs, 22 tRNAs, 2 rRNAs (rrnL and rrnS), and a control region (D-loop) (Figure 1). Among the 37 fragment genes, a PCG (*ND6*) and 8 tRNA genes (tRNA-Ala, tRNA-Cys, tRNA-Glu, tRNA-Gln, tRNA-Asn, tRNA-Pro, tRNA-Ser2, and tRNA-Tyr) were located on the light strand (L-strand), whereas the CR and the remaining 28 genes were located on the heavy strand (H-strand). The gene order was the same as that in most bird mitochondrial genomes [37,38] (Table 2). The total length of protein-coding, tRNA, and rRNA genes comprised 68.65%, 9.29%, and 15.57% of the entire mitochondrial genome, respectively. The base composition of the *A. marila* genome was 29.42% A, 22.25% T, 15.49% G, and 32.84% C, and the A + T content (51.67%) was higher than the G + C content (48.33%), suggesting a strong A + T bias. A relatively high AT content has also been reported in other *Aythya* species [36]. In addition, AT-skews and GC-skews are often used to assess nucleotide-compositional differences in mitochondrial genomes [39]. We found that the *A. marila* mitochondrial genome AT-skew (0.139) was positive, whereas the GC-skew (−0.359) was negative, indicating higher A and C abundances than T and G abundances, respectively (Table 3).

### 3.2. Protein-Coding Genes and Codon Usage

The mitochondrial genome of *A. marila* has 13 coding genes with a total length of 11,407 bp and encodes cytochrome b, 2 ATPases, 3 cytochrome c oxidase subunits, and 6 NADH dehydrogenase subunits. The lengths of the 13 PCGs range from 168 to 1824 bp; among these, 10 were initiated by the start codon ATG, whereas 3 (*COX I*, *COX II*, and *ND5*) were initiated by the start codon GTG. Seven protein genes (*ATP6*, *ATP8*, *COII*, *ND3*, *ND4L*, *ND5*, and *Cytb*) used TAA as the stop codon. For *COI* and *ND1*, the stop codon was AGG, whereas that for *ND6* was TAG. *ND2*, *ND4*, and *COIII* ended with the incomplete stop codon T (Table 2).

**Figure 2:**
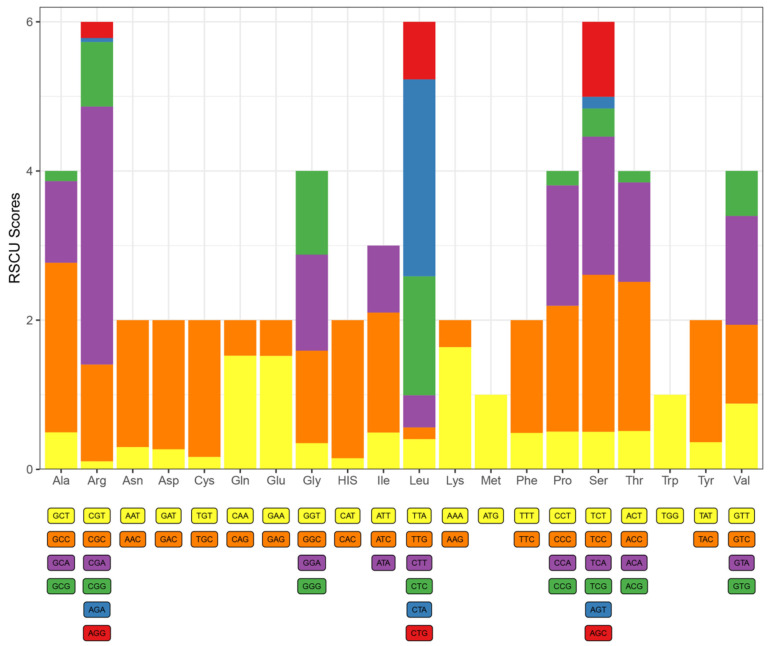
RSCU values of protein-coding genes in the mitochondrial genome of *A. marila*. The *x*-axis denotes the distinct codon families.

The average A + T content of PCGs in the mitochondrial genome of *A. marila* was 50.58%, ranging from 49.34% (*Cytb*) to 52.03% (*ND4*). Except for *ND6*, the base compositions and skewness of the remaining 12 PCGs were relatively similar. Only the percentages of *ND6* T and G were considerably higher than those of the other genes, with a positive GC-skew (Table 3). The usage of amino acids and RSCU values in the PCGs of *A. marila* are summarized in Figure 2 and Table 4. The most frequently used codons (amino acids) were CUA (317), AUC (208), GCC (173), UUC (167), CUC (166), and ACC (156). Except for Trp and Met, all amino acids have their preferred codons, which is a unique feature of *A. marila* compared to other *Aythya* species identified to date [36].

### 3.3. Ribosomal and Transfer RNA Genes

Similar to other *Aythya* species, the *A. marila* mitochondrial genome contained two rRNAs with a total length of 2587 bp, located on the H-strand. The 12S rRNA gene and 16S rRNA are located between tRNA-Phe and tRNA-Leu, which are typically separated by tRNA-Val. The base composition of the 12S rRNA gene was A = 32.62%, T = 19.21%, C = 28.2%, and G = 20.02%, whereas that of the 16S rRNA gene was A = 34.56%, T = 19.90%, C = 25.76%, and G = 19.78%; both rRNAs had a slight AT bias (Table 3).

**Figure 3:**
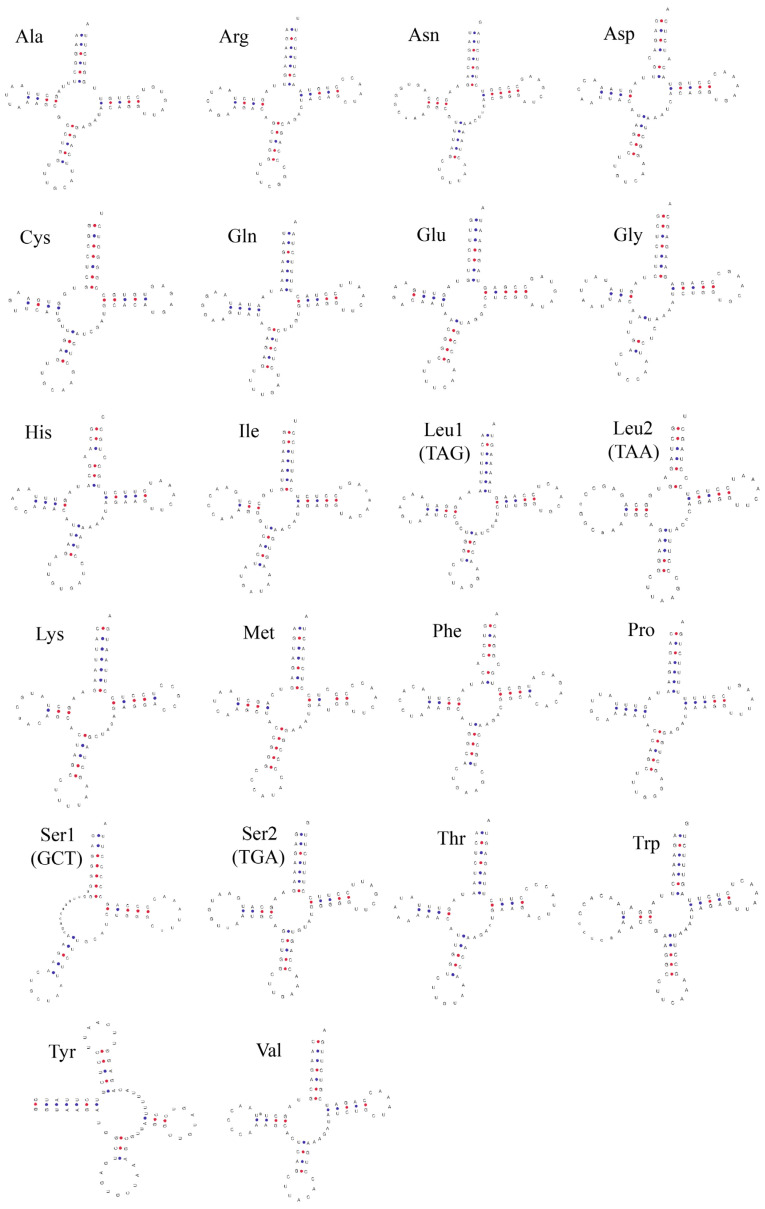
Secondary structures of the tRNA genes in the mitochondrial genome of *A. marila*. Red dots (•) specify G-C base pairing, and purple dots (•) specify G-U or A-U base pairing.

The *A. marila* mitochondrial genome contained 22 tRNAs ranging in size from 66 bp to 76 bp, with an obvious AT bias (56.65%). Of these, 14 were located on the H-strand, and 8 were located on the L-strand (Table 2). The secondary structure of tRNA is shown in Figure 3. In many vertebrate mitochondrial genomes, the tRNA-Ser (GCT) gene has an unusual secondary structure owing to the lack of a dihydrouridine arm. However, some previous studies have shown that this structure does not affect tRNA function [40,41,42,43]; other than tRNA-Ser (GCT), tRNA genes form the classical cloverleaf secondary structure, and the anticodon arms contain a relatively conserved region [40].

### 3.4. Control Region and Repetitive Sequences

**Figure 4:**
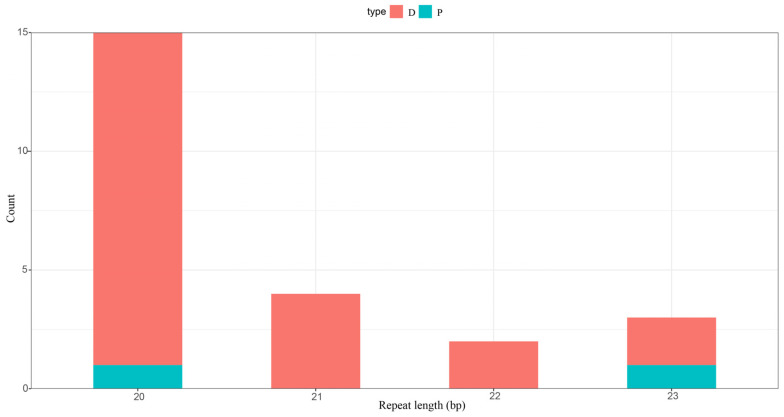
Analyses of repeats in the *A. marila* mitochondrial genome.

A control region was identified in the mitochondrial genome of *A. marila*. This region is located between tRNA-Glu and tRNA-Phe and has a length of 1066 bp. The AT content (51.97%) and GC content (48.03%) of this region were similar to those of the whole mitochondrial genome (Table 3). We also found that the control region AT-skew (0.051) was positive, whereas the GC-skew (−0.340) was negative, which indicated that the A content was higher than the T content and that the G content was lower than the C content. A total of 24 repeat sequences were detected in the *A. marila* mitochondrial genome, which contained 22 forward repeats and 2 palindromic repeats (Figure 4). The repeat sequences ranged from 20 to 23 bp with a total size of 497 bp, constituting 2.99% of the mitochondrial genome.

### 3.5. Synonymous (Ka) and Nonsynonymous (Ks) Substitution Rate

**Figure 5:**
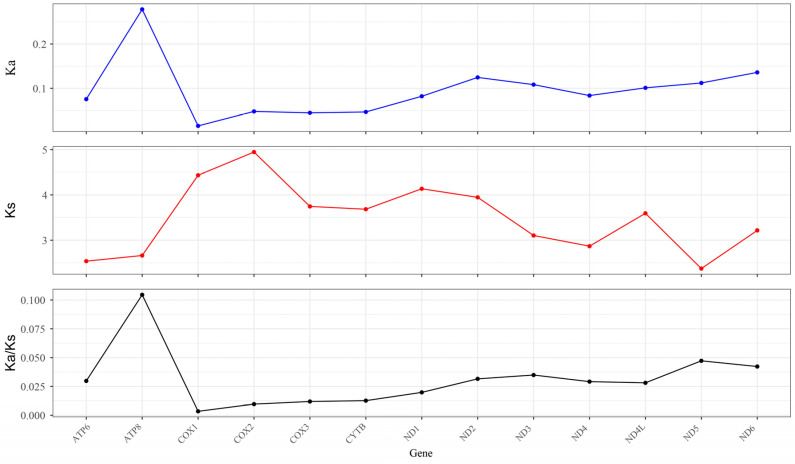
The Ka/Ks ratios of the 13 protein-coding genes of *A. marila*.

The Ka/Ks ratio can be used to determine whether the PCGs were under selection pressure [44,45]. To detect the pressure on *A. marila* mitochondrial PCGs, we calculated the Ka/Ks ratio of the 13 PCGs. As shown in Figure 5, the Ka/Ks ratio did not exceed 0.105 for any genes, indicating that these genes underwent intense purification selection. Moreover, the stabilization of the normal function of mitochondria may be due to the important role of these genes in purification selection [46]. *ATP8* had the highest Ka/Ks value (Ka/Ks = 0.105), indicating that *ATP8* experienced the least selective pressure and evolved the fastest. However, the Ka/Ks value of *COI* was the lowest (Ka/Ks = 0.003), indicating that *COI* had the highest selective pressure and had evolved the slowest.

### 3.6. Population Genetic Diversity and Differentiation

The complete mitochondrial genome sequence was used to estimate the genetic diversity and differentiation of the *Aythya* population. A total of 28 haplotypes were identified from 28 individuals, of which 4 were from *A. nyroca,* with the other 4 *Aythya* species each having 6 haplotypes. The haplotype diversity of the 5 populations was 1, whereas the nucleotide diversity (π) ranged from 0.00080 to 0.01109 per population (Table 5). These results show that our target species had the highest genetic diversity, whereas the critically endangered *A. baeri* had the lowest genetic diversity. The low genetic diversity of *A. baeri* is consistent with its population size, suggesting that it may be at risk of extinction. In addition, there was obvious differentiation between the five populations; however, no significant genetic differentiation was found between *A. baeri* and *A. nyroca* (*F*_ST_ = −0.09740), suggesting that genetic exchange between the two populations is common (Table 6).

### 3.7. Phylogenetic Analysis

**Figure 6:**
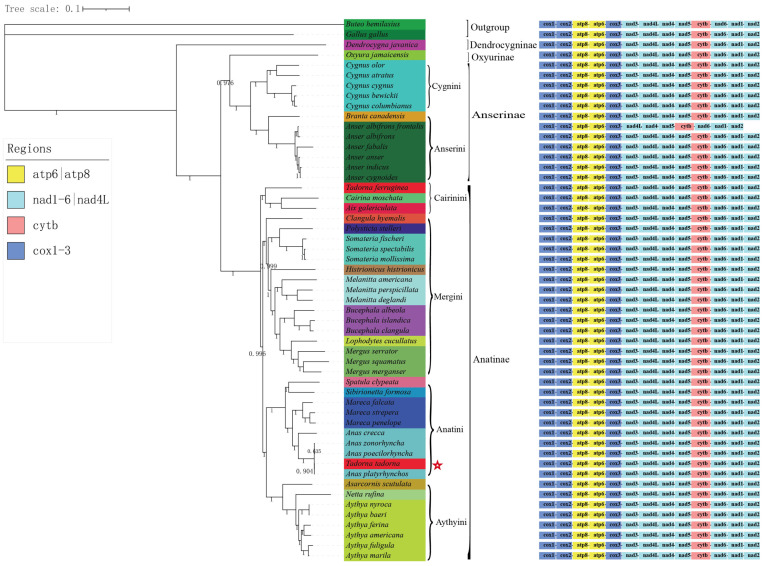
Phylogenetic relationship of Anatidae species based on 13 protein-coding genes. Numbers above branches are posterior probabilities from BI. *B. hemilasius* and *G. gallus* served as outgroup species. The red pentagram indicates that *Todoma todoma* may be a hybrid.

We performed a comprehensive phylogenetic analysis on the mitochondrial genomes of all Anatidae species available in GenBank, employing both Bayesian inference (BI) and maximum likelihood (ML) methodologies. Both approaches yielded high bootstrap support values and Bayesian posterior probabilities. Our findings revealed that the BI and ML phylogenetic trees shared identical topologies; however, the tree generated using BI exhibited superior support values. Consequently, we exclusively present the BI tree in this study (Figure 6). In the phylogenetic tree, there were four major clades among the Anatidae: Dendrocygninae, Oxyurinae, Anserinae, and Anatinae. The first branch, Dendrocygninae, contained only *Dendrocygna javanica*. *D. javanica* constitutes an early diverging lineage, representing one of the most distinctive genera within the Anatidae family. Their unique characteristics, such as an erect posture, elongated necks and legs, and tree-perching habits, distinguish them from the majority of other waterfowl species [47,48].

The second is Oxyurinae, which consists of only *Oxyura jamaicensis*. The phylogenetic relationship of Oxyurinae is also controversial [49]. According to early morphological studies, Oxyurinae shares a common ancestor with geese and swans [49,50], leading us to consider Oxyurinae as the group most closely related to Anserinae whilst not being within Anatinae. In previous studies, Oxyurinae was shown to have diverged earlier than Dendrocygninae in Anatidae [51], and our results also support Oxyurinae as an independent subfamily. The third is Anserinae, which includes Cygnini and Anserini. Anserini contains the genera Anser and Branta, which are closely related species and sisters to the Cygnini assemblage, which is consistent with morphological data [52]. Meigini, Anatini, Aythyini, and Cairinini form the fourth branch of the Anatinae. Within Anatinae, Aythyini is monophyletic, Aythyini and Anatini have the closest phylogenetic relationships, and Aythyini diverged earlier than Anatini based on morphological studies and phylogenetic analysis [49,53]. Within the genera, *Aythya*, *A. marila,* and *A. fuligula* form a branch, whereas *A. americana* and *A. ferina*, and *A. baeri* and *A. nyroca* were clustered into one branch, with all branches being supported by good statistical values. In general, our findings support those of the previous molecular phylogenetic studies.

**Figure 7:**
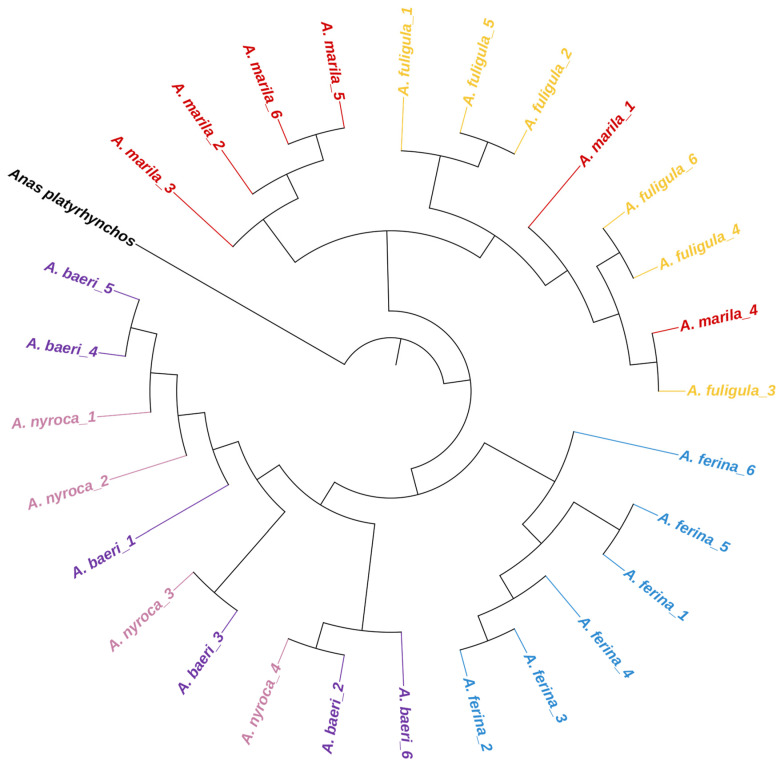
Population structure of *Aythya* species. *A. platyrhynchos* function as outgroup species for comparative analysis.

In an effort to further elucidate the population structure among the five *Aythya* species (*A. ferina*, *A. nyroca*, *A. baeri*, *A. fuligula*, and *A. marila*), we conducted a comprehensive phylogenetic analysis based on complete mitochondrial genomes (Figure 7). Our findings revealed that the *Aythya* genus can be classified into three distinct clades: one comprising *A. baeri* and *A. nyroca*, which exhibit a relatively close phylogenetic relationship; another encompassing *A. marila* and *A. fuligula*, which are also closely related; a third, separate clade consisting solely of *A. ferina*. In addition, our results demonstrated that *A. baeri* and *A. nyroca* display evident mixed clustering patterns, while *A. marila* and *A. fuligula* exhibit an interwoven arrangement, suggesting the potential for frequent hybridization events within the *Aythya* genus. However, it is important to note that the maternal inheritance characteristics of the mitochondrial genome, coupled with the pervasive hybridization behavior observed in the *Aythya* genus, may introduce inaccuracies when attempting to depict the population structure of these species.

## 4. Conclusions

In the present study, the complete mitochondrial genome of *A. marila* was sequenced, annotated, and reported for the first time. The mitochondrial genome sequence of *A. marila* was 16,617 bp in length and consisted of 13 PCGs, 2 rRNA genes, 22 tRNA genes, and a D-loop. Among the PCGs, *ATP8* evolved the fastest, whereas *COI* evolved the slowest. Phylogenomic analysis based on 13 protein-coding genes among 52 Anatidae species showed that *A. marila* was closely related to *A. fuligula*. We also assembled 28 complete mitochondrial genomes of 5 *Aythya* species and found no significant genetic differentiation between *A. baeri* and *A. nyroca*. Moreover, *A. baeri* had the lowest genetic diversity, which not only complemented the available *Aythya* species resources but also further supported their taxonomic status. Our results also provide a beneficial reference for the taxonomy, population genetics, and systematic studies of *Aythya* species.

## Figures and Tables

**Table 1:** Twenty-eight samples information used in the study.

Species	Number	Sample Collection Site
*A. marila*	6	Heilongjiang Province, China
*A. ferina*	6	Heilongjiang Province, China
*A. nyroca*	4	Heilongjiang Province, China
*A. baeri*	6	Heilongjiang Province, China
*A. fuligula*	6	Heilongjiang Province, China

**Table 2:** Organization of the *A. marila* mitochondrial genome.

Gene	Strand	Location	Size (bp)	Intergenic Length	Anticodon	Start Codon	Stop Codon
tRNA-Phe	H	1–70	70	0	GAA	-	-
12S rRNA	H	71–1054	984	0	-	-	-
tRNA-Val	H	1055–1125	71	0	TAC	-	-
16S rRNA	H	1126–2728	1603	0	-	-	-
tRNA-Leu2	H	2729–2802	74	4	TAA	-	-
*ND1*	H	2807–3784	978	−2	-	ATG	AGG
tRNA-Ile	H	3783–3854	72	8	GAT	-	-
tRNA-Gln	L	3863–3933	71	−1	TGG	-	-
tRNA-Met	H	3933–4001	69	0	CAT	-	-
*ND2*	H	4002–5040	1039	0	-	ATG	T--
tRNA-Trp	H	5041–5116	76	3	TCA	-	-
tRNA-Ala	L	5120–5188	69	2	TGC	-	-
tRNA-Asn	L	5191–5263	73	0	GTT	-	-
tRNA-Cys	L	5264–5329	66	0	GCA	-	-
tRNA-Tyr	L	5330–5399	70	1	GTA	-	-
*COI*	H	5401–6951	1551	−9	-	GTG	AGG
tRNA-Ser2	L	6943–7015	73	2	TGA	-	-
tRNA-Asp	H	7018–7086	69	1	GTC	-	-
*COII*	H	7088–7774	687	1	-	GTG	TAA
tRNA-Lys	H	7776–7843	68	1	TTT	-	-
*ATP8*	H	7845–8012	168	−10	-	ATG	TAA
*ATP6*	H	8003–8686	684	−1	-	ATG	TAA
*COIII*	H	8686–9469	784	0	-	ATG	T--
tRNA-Gly	H	9470–9538	69	0	TCC	-	-
*ND3*	H	9539–9890	351	1	-	ATG	TAA
tRNA-Arg	H	9892–9961	70	0	TCG	-	-
*ND4L*	H	9962–10,258	297	−7	-	ATG	TAA
*ND4*	H	10,252–11,629	1378	0	-	ATG	T--
tRNA-His	H	11,630–11,698	69	0	GTG	-	-
tRNA-Ser1	H	11,699–11,764	66	−1	GCT	-	-
tRNA-Leu1	H	11,764–11,834	71	0	TAG	-	-
*ND5*	H	11,835–13,658	1824	−1	-	GTG	TAA
*Cytb*	H	13,658–14,800	1143	2	-	ATG	TAA
tRNA-Thr	H	14,803–14,871	69	10	TGT	-	-
tRNA-Pro	L	14,882–14,951	70	10	TGG	-	-
*ND6*	L	14,962–15,483	522	0	-	ATG	TAG
tRNA-Glu	L	15,484–15,551	68	0	TTC	-	-
D-loop	H	15,552–16,617	1066	0	-	-	-

**Table 3:** Nucleotide composition and skewness of *A. marila* mitochondrial genome.

*A. marila*	T%	C%	A%	G%	(A + T)%	AT-Skew	GC-Skew	Length (bp)
Mitogenome	22.25	32.84	29.42	15.49	51.67	0.139	−0.359	16,617
PCGs	23.56	33.98	27.02	15.44	50.58	0.068	−0.375	11,407
*COI*	24.11	32.95	25.98	16.96	50.09	0.037	−0.320	1551
*COII*	22.12	33.19	27.66	17.03	49.75	0.111	−0.315	687
*ATP8*	17.86	40.47	33.93	7.74	51.79	0.310	−0.679	168
*ATP6*	23.39	37.99	27.92	11.70	51.31	0.088	−0.540	684
*COIII*	23.09	33.67	26.91	16.33	50.00	0.076	−0.347	784
*ND3*	23.55	34.42	26.81	15.22	50.36	0.065	−0.387	351
*ND5*	21.32	35.09	29.99	13.60	51.31	0.169	−0.441	1824
*ND4*	22.71	36.00	29.32	11.97	52.03	0.127	−0.501	1378
*ND4L*	24.58	33.67	25.59	16.16	50.17	0.020	−0.351	297
*ND6*	39.46	11.88	10.15	38.51	49.61	−0.591	0.528	522
*Cytb*	23.01	36.31	26.33	14.35	49.34	0.067	−0.433	1143
*ND1*	24.34	34.66	25.36	15.64	49.70	0.021	−0.378	978
*ND2*	21.75	36.48	29.55	12.22	51.30	0.152	−0.498	1039
D-loop	24.67	32.18	27.30	15.85	51.97	0.051	−0.340	1066
tRNAs	26.64	22.03	30.01	21.32	56.65	0.059	−0.016	1543
12S rRNA	19.21	28.25	32.62	20.02	51.83	0.259	−0.171	984
16S rRNA	19.90	25.76	34.56	19.78	54.46	0.269	−0.131	1603

**Table 4:** The codon number and relative synonymous codon usage in the mitochondrial genome of *A. marila*.

Codon	Count	RSCU	Codon	Count	RSCU	Codon	Count	RSCU	Codon	Count	RSCU
UUU(F)	52	0.475	UCU(S)	23	0.491	UAU(Y)	18	0.340	UGU(C)	2	0.148
UUC(F)	167	1.525	UCC(S)	100	2.135	UAC(Y)	88	1.660	UGC(C)	27	1.852
UUA(L)	41	0.373	UCA(S)	87	1.858	UAA(*)	7	-	UGA(*)	87	-
UUG(L)	13	0.118	UCG(S)	13	0.278	UAG(*)	1	-	UGG(W)	19	1
CUU(L)	46	0.419	CCU(P)	25	0.435	CAU(H)	12	0.216	CGU(R)	1	0.081
CUC(L)	166	1.511	CCC(P)	94	1.635	CAC(H)	99	1.784	CGC(R)	16	0.297
CUA(L)	317	2.887	CCA(P)	102	1.774	CAA(Q)	67	1.489	CGA(R)	45	3.649
CUG(L)	76	0.692	CCG(P)	9	0.156	CAG(Q)	23	0.511	CGG(R)	10	0.811
AUU(I)	65	0.508	ACU(T)	31	0.392	AAU(N)	16	0.264	AGU(S)	7	0.241
AUC(I)	208	1.625	ACC(T)	156	1.975	AAC(N)	105	1.736	AGC(S)	51	1.759
AUA(I)	111	0.867	ACA(T)	115	1.456	AAA(K)	77	1.730	AGA(R)	0	0
AUG(M)	58	1	ACG(T)	14	0.177	AAG(K)	12	0.270	AGG(R)	2	0.162
GUU(V)	37	0.751	GCU(A)	40	0.559	GAU(D)	8	0.267	GGU(G)	11	0.204
GUC(V)	61	1.239	GCC(A)	173	2.420	GAC(D)	52	1.733	GGC(G)	77	1.426
GUA(V)	72	1.462	GCA(A)	91	1.273	GAA(E)	72	1.532	GGA(G)	74	1.370
GUG(V)	27	0.548	GCG(A)	12	0.168	GAG(E)	22	0.468	GGG(G)	54	1

* represents the termination codon.

**Table 5:** Genetic diversity of five *Aythya* species.

Species	N_H_	N	h	π
*A. marila*	6	6	1	0.01109
*A. ferina*	6	6	1	0.00139
*A. nyroca*	4	4	1	0.00093
*A. baeri*	6	6	1	0.00080
*A. fuligula*	6	6	1	0.00087

N_H_, number of haplotypes; N, number of individuals; h, haplotype diversity; π, nucleotide diversity.

**Table 6:** Fixation index (*F*_ST_) of five *Aythya* species.

Species	*A. fuligula*	*A. marila*	*A. ferina*	*A. nyroca*
*A. marila*	0.55425			
*A. ferina*	0.96521	0.78815		
*A. nyroca*	0.97498	0.82212	0.96901	
*A. baeri*	0.97628	0.82312	0.97023	−0.09740

## Data Availability

The complete mitochondrial genome sequence of *A. marila* has been submitted to GenBank with the accession number OP326610. The 28 complete mitochondrial genomes of five species: six *A. ferina*, four *A. nyroca*, six *A. baeri*, six *A. fuligula*, and six *A. marila* were deposited in SRA under the following accession numbers: SRR24490231, SRR24488788, SRR24489043, SRR24490229 and SRR24489013.
